# Efficacy and safety of renal denervation therapy in hypertension: a meta-analysis

**DOI:** 10.1093/ehjopen/oeaf026

**Published:** 2025-03-13

**Authors:** Husam M Salah, Jorge Antonio Gutierrez, Jennifer A Rymer, Hidenori Yaku, Rajesh V Swaminathan, Manesh R Patel, Marat Fudim

**Affiliations:** Division of Cardiology, Department of Medicine, Duke University, 2301 Erwin Rd, Durham, NC 27710, USA; Division of Cardiology, Department of Medicine, Duke University, 2301 Erwin Rd, Durham, NC 27710, USA; Division of Cardiology, Department of Medicine, Duke University, 2301 Erwin Rd, Durham, NC 27710, USA; Duke Clinical Research Institute, 300 W Morgan St, Durham, NC 27701, USA; Division of Cardiology, Northwestern University Feinberg School of Medicine, 676 North St. Clair St, Chicago, IL 60611, USA; Division of Cardiology, Department of Medicine, Duke University, 2301 Erwin Rd, Durham, NC 27710, USA; Duke Clinical Research Institute, 300 W Morgan St, Durham, NC 27701, USA; Division of Cardiology, Department of Medicine, Duke University, 2301 Erwin Rd, Durham, NC 27710, USA; Duke Clinical Research Institute, 300 W Morgan St, Durham, NC 27701, USA; Division of Cardiology, Department of Medicine, Duke University, 2301 Erwin Rd, Durham, NC 27710, USA; Duke Clinical Research Institute, 300 W Morgan St, Durham, NC 27701, USA

Although randomized sham-controlled trials have shown an overall numerical reduction in blood pressure with renal denervation therapy (RDN) in patients with hypertension, there has been inconsistency in this effect and its magnitude in these trials, often not achieving statistical significance, mainly due to the relatively small sample size of these trials. In addition, early experience with the first-generation RDN catheters, which consist of a single unipolar electrode, suggested that incomplete circumferential ablation may attenuate the blood pressure lowering effect of the procedure and, subsequently, led to the development of the second-generation RDN devices, which allow for a more circumferential ablation sites.^1,2^ Most recently, the TARGET BP I trial found a statistically significant reduction in 24 h ambulatory systolic blood pressure (SBP) with alcohol-mediated RDN, which allows for 360° spread of alcohol.^3^ Herein, we aim to assess the effect and safety of RDN and the magnitude of its effect on blood pressure using a meta-analytic approach.

PubMed/Medline was searched from inception until 8 March 2024, using the following terms: (‘renal denervation’ and ‘hypertension’). The search was restricted to randomized clinical trials (RCTs) using the advanced search tool. The pre-specified selection criteria were: (i) RCTs using RDN in the active arm for treatment of hypertension and sham in the control group; and (ii) pre-specified efficacy and safety endpoints were reported in the trials or can be calculated. Efficacy endpoints were mean change in 24 h ambulatory SBP and office SBP. The safety endpoint was the composite of all-cause death, stroke, myocardial infarction, worsening kidney function, hospitalization for hypertensive crisis, or major access-site complications. When an endpoint was analysed at multiple time points within a trial, the one with the longest follow-up was included. If a study did not report all components of the composite safety endpoint, we included only the available components in the analysis. Two investigators (H.M.S. and M.F.) conducted the study search, selection, and data abstracting. The inverse variance of weighted mean difference and associated 95% confidence intervals (CIs) were used to assess changes in 24 h ambulatory and office SBPs. Mantel–Haenszel risk ratios (RRs) and the associated 95% CIs were used to assess the safety endpoint. A random-effect model meta-analysis was performed. Heterogeneity was assessed using Cochrane *Q* statistic, and Higgins and Thompsons’ *I*^2^. A subgroup analysis was performed based on continuing vs. withholding antihypertensive agents during the study period. Sensitivity analyses were performed by excluding trials of first-generation RDN devices (i.e. devices with a single unipolar electrode) and based on the modality of RDN. Review Manager 5.4 was used to conduct all analyses.

Twelve RCTs with a total of 1926 patients (RDN: 1100, sham: 826) were included. Five RCTs stopped antihypertensive medications prior to the study period, whereas the rest continued them. Follow-up period ranged from 2 to 36 months a median of 4.5 months. Renal denervation resulted in a significant reduction in 24 h ambulatory SBP [mean difference −4.53 mmHg; 95% CI (−7.50, −1.57); *I*^2^ = 80%] and office SBP [mean difference −6.67 mmHg; 95% CI (−10.96, −2.39); *I*^2^ = 81%]. When pooling data from the trials that continued antihypertensive agents, there was a statistically significant reduction in 24 h ambulatory SBP [mean difference −5.60 mmHg; 95% CI (−10.12, −1.09); *I*^2^ = 85%] and office SBP [mean difference −8.48 mmHg; 95% CI (−16.39 −0.56); *I*^2^ = 90%]. When pooling data from the trials that withheld antihypertensive agents, there was a statistically significant reduction in office SBP [mean difference −4.84 mmHg; 95% CI (−7.92, −1.75); *I*^2^ = 26%] but no significant reduction in 24 h ambulatory SBP [mean difference −2.96; 95% CI (−6.82, 0.91); *I*^2^ = 71%]. Sensitivity analysis by excluding trials of first-generation RDN devices showed consistent results of the primary efficacy endpoint. In a sensitivity analysis based on the modality of RDN, radiofrequency (RF) and endovascular ultrasound (US) RDN modalities significantly reduced both 24 h ambulatory SBP [RF: mean difference −7.22 mmHg; 95% CI (−12.96, −1.48); *I*^2^ = 81% vs. endovascular US: mean difference −4.34 mmHg; 95% CI (−6.46, −2.21); *I*^2^ = 27%] and office SBP [RF: mean difference −12.61 mmHg; 95% CI (−22.51, −2.72); *I*^2^ = 81% vs. endovascular US: mean difference −5.41 mmHg; 95% CI (−7.81, −3.00); *I*^2^ = 0%]. Alcohol-mediated RDN demonstrated no effects on 24 h ambulatory SBP [mean difference 0.75 mmHg; 95% CI (−7.56, 9.06); *I*^2^ = 86%] or office SBP [mean difference −1.12 mmHg; 95% CI (−6.02, 3.77); *I*^2^ = 41%].

Across all included studies, there was no significant difference in the composite safety endpoint between RDN and sham [RR 1.05; 95% CI (0.70, 1.56); *I*^2^ = 0%; *Figure 1*]. Due to the limited number of safety events reported across trials and the potential for misleading conclusions, we did not perform subgroup or sensitivity analyses for the safety endpoint.

**Figure 1:**
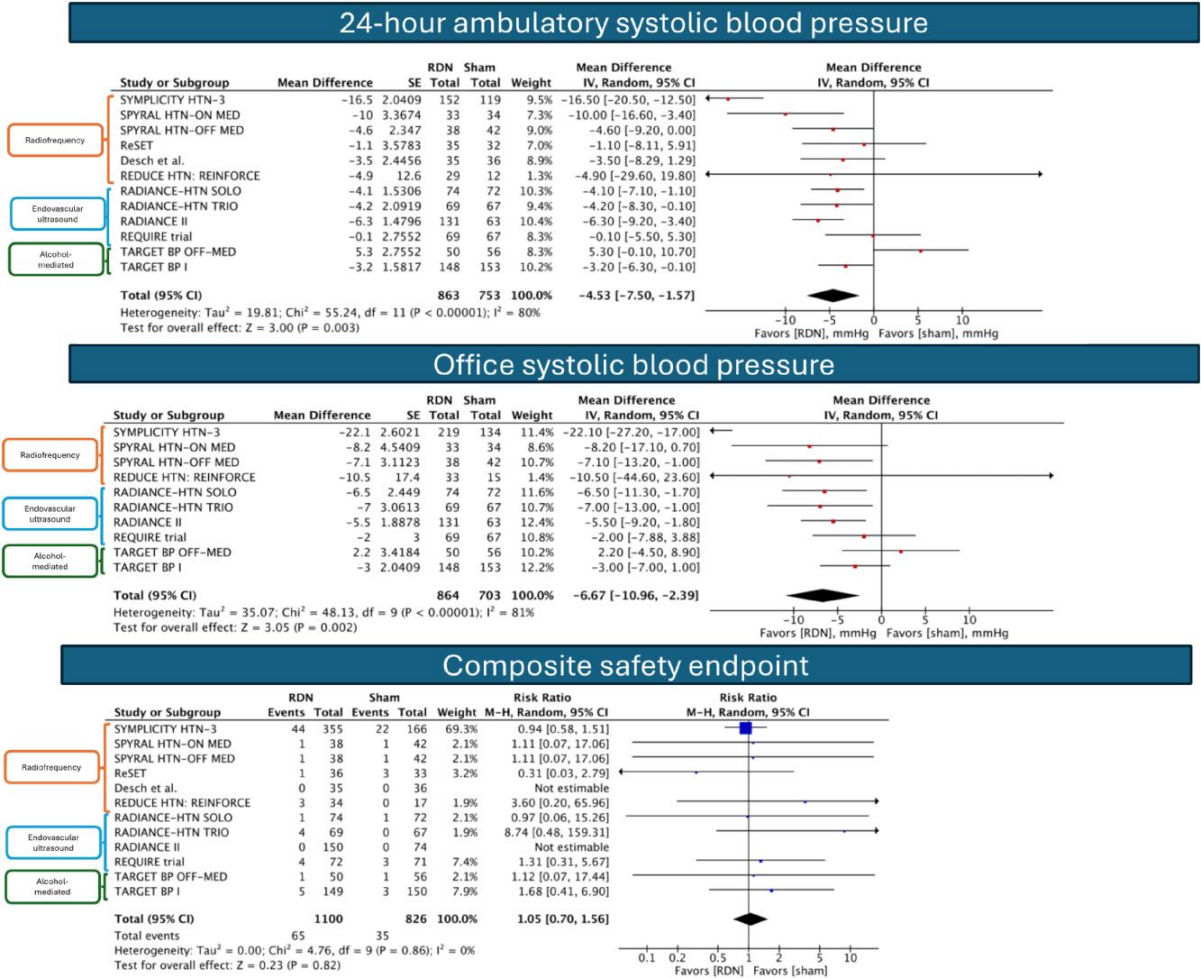
Forest plots showing the efficacy and safety endpoints.

This meta-analysis shows that RDN can result in incremental benefits in patients with hypertension with a 4.5 mmHg mean reduction in 24 h ambulatory SBP and a 6.7 mmHg mean reduction in office SBP with a signal of a better effect on a background of antihypertensive agents. Whether this modest reduction in blood pressure can translate into improvement in clinical outcomes needs to be studied in future clinical trials. However, this meta-analysis demonstrated marked statistical heterogeneity in the primary efficacy endpoints, which is an important limitation. Trials continuing antihypertensive medications exhibited higher heterogeneity compared with those withholding medications. Blood pressure reductions were statistically significant for office SBP in both subgroups, but for 24 h ambulatory SBP, a significant reduction was only observed in trials continuing antihypertensive therapy. This indicates that background antihypertensive therapy may influence the magnitude of the effect, but it is likely one of several contributors to the observed heterogeneity. Other potential contributors to heterogeneity include differences in patient populations, trial designs, and follow-up durations. This variability may have influenced the overall effect estimates, making it difficult to determine the precise impact of RDN across different clinical scenarios.

Despite the overall favourable safety profile observed in this analysis, the number of safety events was low across studies, especially in specific RDN modalities. This limits the ability to draw firm conclusions regarding the comparative safety of different techniques. Also, as not all trials reported every component of the composite safety endpoint, variations in event reporting could have influenced our pooled estimates. This limitation should be considered when interpreting our safety findings. Future studies with larger sample sizes and longer follow-up are needed to better assess the long-term safety and efficacy of RDN.

Despite the limitations of this meta-analysis [i.e. study-level meta-analysis of published summary data rather than individual patient level data, different RDN methods (RF, endovascular US, and alcohol-mediated), marked heterogeneity in the primary efficacy endpoints, and variable follow-up periods], it suggests that RDN is a promising effective and safe avenue to address hypertension, which is a major risk factor for cardiovascular diseases that are becoming the leading causes of hospitalization, mortality, and morbidity worldwide.^4,5^

## Data Availability

The data underlying this article will be shared on reasonable request to the corresponding author.

## Lead author biography

Husam M. Salah, MD, is a second-year cardiology fellow at Duke University Medical Center. He earned his medical degree from Jordan University of Science and Technology and completed his internal medicine residency at the University of Arkansas for Medical Sciences, where he also served as chief resident. His research focuses on the intersection of interventional cardiology and heart failure, with a particular interest in device-based cardiovascular interventions.

## References

[1] Kandzari DE, Bhatt DL, Brar S, Devireddy CM, Esler M, Fahy M, Flack JM, Katzen BT, Lea J, Lee DP, Leon MB, Ma A, Massaro J, Mauri L, Oparil S, O’Neill WW, Patel MR, Rocha-Singh K, Sobotka PA, Svetkey L, Townsend RR, Bakris GL. Predictors of blood pressure response in the SYMPLICITY HTN-3 trial. Eur Heart J 2014;36:219–227. 25400162 10.1093/eurheartj/ehu441 PMC4301597

[2] Versaci F, Sciarretta S, Scappaticci M, Calcagno S, di Pietro R, Sbandi F, Dei Giudici A, Del Prete A, de Angelis S, Biondi-Zoccai G. Renal arteries denervation with second generation systems: a remedy for resistant hypertension? Eur Heart J Suppl 2020;22:L160–L165. 33239993 10.1093/eurheartj/suaa158 PMC7673618

[3] Kandzari DE, Weber MA, Pathak A, Zidar JP, Saxena M, David SW, Schmieder RE, Janas AJ, Langer C, Persu A, Mendelsohn FO, Ameloot K, Foster M, Fischell TA, Parise H, Mahfoud F. Effect of alcohol-mediated renal denervation on blood pressure in the presence of antihypertensive medications: primary results from the TARGET BP I randomized clinical trial. Circulation 2024;149:1875–1884. 38587557 10.1161/CIRCULATIONAHA.124.069291

[4] Mills KT, Stefanescu A, He J. The global epidemiology of hypertension. Nat Rev Nephrol 2020;16:223–237. 32024986 10.1038/s41581-019-0244-2 PMC7998524

[5] Salah HM, Minhas AMK, Khan MS, Pandey A, Michos ED, Mentz RJ, Fudim M. Causes of hospitalization in the USA between 2005 and 2018. Eur Heart J Open 2021;1:oeab001. 35919090 10.1093/ehjopen/oeab001 PMC9242058

